# flEECe, an energy use and occupant behavior dataset for net-zero energy affordable senior residential buildings

**DOI:** 10.1038/s41597-019-0275-3

**Published:** 2019-11-26

**Authors:** Frederick Paige, Philip Agee, Farrokh Jazizadeh

**Affiliations:** 1 Charles E. Via Jr. Department of Civil and Environmental Engineering, Virginia Tech, Blacksburg, VA 24061 United States; 2 Myers-Lawson School of Construction, Virginia Tech, Blacksburg, VA 24061 United States

**Keywords:** Energy and behaviour, Energy efficiency

## Abstract

The behaviors of building occupants have continued to perplex scholars for years in our attempts to develop models for energy efficient housing. Building simulations, project delivery approaches, policies, and more have fell short of their optimistic goals due to the complexity of human behavior. As a part of a multiphase longitudinal affordable housing study, this dataset represents energy and occupant behavior attributes for 6 affordable housing units over nine months in Virginia, USA which are not performing to the net-zero energy standard they were designed for. This dataset provides researchers the ability to analyze the following variables: energy performance, occupant behaviors, energy literacy, and ecological perceptions. Energy data is provided at a 1 Hz sampling rate for four circuits: main, hot water heater, dryer, and HVAC. Building specifications, occupancy, weather data, and neighboring building energy use data are provided to add depth to the dataset. This dataset can be used to update building energy use models, predictive maintenance, policy frameworks, construction risk models, economic models, and more.

## Abstract (summary)

**Table:** 

Measurement(s)	electrical energy • occupant energy literacy • atmospheric weather • climate
Technology Type(s)	Monitoring Device • Gauge or Meter Device • survey method • digital curation
Sample Characteristic - Environment	residential building
Sample Characteristic - Location	Commonwealth of Virginia

Machine-accessible metadata file describing the reported data: 10.6084/m9.figshare.9891995

## Background & Summary

Building occupant education is an important research direction providing an opportunity to maximize the efficacy of energy efficiency investments in the housing industry^1^. Despite increasing efforts by property managers to educate tenants on technology in housing units, many residents lack an understanding of energy efficient technologies. As technology becomes further integrated into housing units, user education will become more of a factor in the optimization of residential energy use. For example, McCoy et. al. reported that residents that received education on their apartments had a lower average energy usage monthly and annually (over 3 years) by almost 15% (14.8%) and a lower energy bill by $10.56 per month^2^.

Providing an education to residents is not only the appropriate ethical choice, but also it can be a powerful business strategy for builder-developers. In the context of this dataset, electricity pricing in Virginia is trending upwards with United States and global rates. Virginia electricity prices have increased by an average of 1.5% per year over 25 years and 3% annually over the last ten years^3^. Traditionally, utilities provide education in the form of energy usage feedback with a “lag” which becomes more detrimental as prices rise. Human factors researchers have reported that people are generally poor at managing systems with lags in information and delayed feedback loops^4,5^. For energy efficient housing systems, energy monitoring systems exist which can report energy use in real-time using an Energy Feedback Display (EFD) for occupants (meetnexi.com).

Using the same hardware, building managers can utilize cloud-based software platforms to monitor and create performance reporting, instantly being aware of system failures or inefficiencies. Studies have shown that EFDs have improved behavior towards a more energy efficient lifestyle and resulted in 10–15% monthly energy use reductions given: (a) frequent feedback, (b) provided over long periods of time, (c) with appliances characterized individually, (d) presented in clear, appealing ways, and (e) utilizing computerized, interactive tools^6–9^.

In this paper, we release our dataset flEECe^10^. This dataset is the outcome of a unique case study within a longitudinal multiphase study to explore the impacts of feedback and education on energy-use behaviors of seniors in affordable housing units. The units were designed to be net-zero energy and followed the EarthCraft Multifamily design guidelines^11^, adhering to the Low-Income Housing Tax Credit requirements^12^. The participants, in this study, participated in an educational workshop and were provided with a budget-based (giving feedback based on remaining budget) energy monitoring system to receive feedback on their electricity usage in real-time. The following two questions motivated the collection of the presented data.

- How does targeted energy education and energy consumption feedback impact energy literacy of seniors in net-zero energy apartment units?
- How does targeted energy education and energy consumption feedback impact energy consumption of seniors in net-zero energy apartment units?

The use of “how” in the questions reflects the qualitative portion of this study which is not presented in this dataset. The dataset described here is solely the quantitative portion of this mixed methods case study. The quantitative data collected can be analyzed in multiple ways to expand beyond the original objectives of our study.

How occupants shift their energy use behaviors is linked to their environmental perceptions, previous energy behaviors, and level of education^13,14^. Targeted occupant education has shown potential to be an effective method for reducing energy consumption^15,16^ and in a previous phase of this study, education strongly correlated with reduced energy consumption^2^. A unique aspect in our study is the focus on senior residents who are not financially incentivized to conserve energy – a topic which needs further investigation^17^ and is critical in the context of affordable housing. Furthermore, this dataset reflects a case study framed according to the concepts in the U.S. Department of Energy (DOE) Energy Literacy guide^18^ through coded survey responses.

Scholars may use the flEECe dataset to investigate:

- Energy use behaviors in the context of the US DOE Energy literacy concepts
- Long-term resident consumption patterns
- Evaluate the performance of energy efficient water-heaters, mini-splits, and dryers in affordable senior housing
- Understand the impact of climate on consumption
- Non-intrusive energy monitoring techniques for data with different resolution

## Methods

### Case study context

This dataset was collected in the context of a singular case study in continuation of a multiphase state-wide longitudinal study in Virginia^1^. Building upon findings in the last phase of the longitudinal study, this case study utilizes interviews, field observations, surveys, and energy consumption data to explore the impact of targeted education on residents’ energy consumption. The dataset presented in this paper has detailed information on participants energy consumption, perceived behavior, beliefs, and energy literacy. This dataset is an addition to the increased amount of building monitoring data we need for improving residential energy use through advanced metering and monitoring^19,20^.

The property under examination is a senior living Low Income Housing Tax Credit (LIHTC) project in Richmond, Virginia that has been being monitored since 2013. This property was selected for its unique occupant energy consumption patterns and design features. A unique feature of this study pertains to that fact that the occupants are not financially incentivized to conserve energy given that the energy bills in these buildings are paid by the property managers. While the property was performing better than average building nationally and in Virginia, the property was falling short of its net-zero energy goal^2^. As reflected in Tables 1 and 2, the one-bedroom housing units were designed to be grid-tied net-zero housing units with all electric energy efficient appliances, tight building envelope, energy efficient glazing, and solar PV arrays on site. The windows and their shadings are manually operable and there is no known outside factors (such as noise or air pollution) to prevent occupants from opening the windows. Building characteristics can be found within the dataset in the tabular data excel file. A color coded illustration of the building and unit layout, material properties, window to wall ratio, and heating and cooling specifications can be found on the “Architectural Data” tab of the dataset^10^. The 6 units which are the focal point of this study come together to form a 7-unit building. Although all 7 units were monitored, due to unreliable data from one unit, the flEECe dataset only includes circuit level data from 6 units. Also provided in the dataset is monthly data for the adjacent 32-unit building which is a part of the same housing property.

**Table 1:** Building specifications. All electric 1-bedroom units.

Characteristics	Building 1	Building 2
Climate zone	US 4A	US 4A
# of apartments	7 (6 were presented in flEECe)	32
# of buildings	1	1
PV-system	16.9 m^2^, south-facing, 2.43 kWp	166.1 m², south-facing, 2.43 kWp
Living area	668 ft^2^ (62.1 m^2^)	708 ft² (65.9 m^2^)
Building volume	6,178 cf (175 m^3^)	6,372 cf (180 m^3^)
Heating system	Air-source heat pump 18 kBtuh, 9 HSPF	Air-source heat pump 18 kBtuh, 9 HSPF
Cooling system	Air-source heat pump, 18 SEER	Air-source heat pump, 18 SEER
Distribution	2, Ductless air systems	2, Ductless air systems
Water heating	Standard electric storage (0.92 EF)	Standard electric storage (0.92 EF)
Ventilation	Energy recovery ventilator	Energy recovery ventilator
Windows U-value SHGC (g-value)	0.30 BTUh/ft^2^/°F (0.95/W/m^2^K) 0.28	0.30 BTUh/ft^2^/°F (0.95/W/m^2^K) 0.28
Wall U-value	0.04 BTUh/ft^2^/°F (0.13/W/m^2^K)	0.04 BTUh/ft^2^/°F (0.13/W/m^2^K)
Roof/Attic U-value	0.03 BTUh/ft^2^/°F (0.11/W/m^2^K)	0.03 BTUh/ft^2^/°F (0.11/W/m^2^K)
Air tightness	4.1 ACH_50_	6.1 ACH_50_

**Table 2:** Behavioral data sample summary.

Meta Data	Total Units	Targeted Education (TEI)	TEI + In-home Display	Control
Number of Units	38	20	6	18
Energy Data	Main aggregate data per month	Main aggregate data per month	Main aggregate data, 3 sub-circuits (dryer, water heater, and air conditioning) – 1-sec, 1-min, and 15-min resolution	Main aggregate data per month
Survey Data		Pre and Post intervention energy perceptions, behaviors, and literacy	Pre and Post intervention energy perceptions, behaviors, and literacy	

### Human data statement

The study protocol used for this study was approved by the Virginia Tech Institutional Review Board. Participants of this study were not subject to any known risks and informed consent was provided from all subjects.

### Energy monitoring protocol

This dataset includes energy use data, collected by advanced circuit level energy monitors and standard utility energy meters. The NEXI energy feedback device^6^ captured second level data (i.e., @1 Hz) for 6 units in this study on four circuits: main, hot-water heater, dryer, and HVAC (mini-split) system using 100A non-invasive split core current transformer clamps with ±1% accuracy. NEXI uses a proprietary data collection and aggregation process designed to work with their unique color-wheel display that provided users with simplified feedback. The detailed energy data from NEXIs including power, voltage, current, and phase angle for four circuits are included in the dataset as raw CSV files. The calibration process for the NEXI data is provided in the dataset as well. Each unit was sub-metered and energy use data was collected per month through a utility benchmark tracker, WegoWise (wegowise.com), which logged five years of monthly energy consumption data for the property. Paper utility bills were used to spot check the data collected by both the NEXI devices and WegoWise platform.

Given the targeted community for this study, we opted for a hard-copy survey which was later digitized for data analysis. Participants were surveyed on site in a community room typically used for social events at the property. The survey combined instruments from literature to measure energy behaviors, perceptions, and literacy^2,21,22^ adapted for use in this case. Surveying in person requires a great deal of flexibility and effort. Surveys were designed to be senior-friendly utilizing large fonts, high contrast, and large formatting for recording responses. Even so, multiple participants needed assistance while taking the survey. A team of two researchers was present to administer the survey with one researcher reading the questions aloud to the group and the other researcher assisting participants one on one when necessary. Property managers helped with recruitment and provided logistics during the education and survey data collection. A $25 gift card was provided by the research team during the pre and post survey, to participating residents to aid in recruitment. The survey questions were coded to the energy literacy guide providing descriptive data on the lessons learned by the study participants.

The recruitment efforts resulted in twenty residents attending a one-hour community meetings and receiving a Tenant Education Intervention (TEI). Of the twenty residents, twenty successful records were developed for analysis. Through stratified sampling, six residents who received the TEI, also were provided with an in-home display. It is important to note that the researchers were able to leverage the existing energy use data from previous work to compare monthly energy usage and the efficacy of resident education efforts. An overview of the behavioral data sample is provided in Table 2.

### Educational intervention

**Fig. 1:**
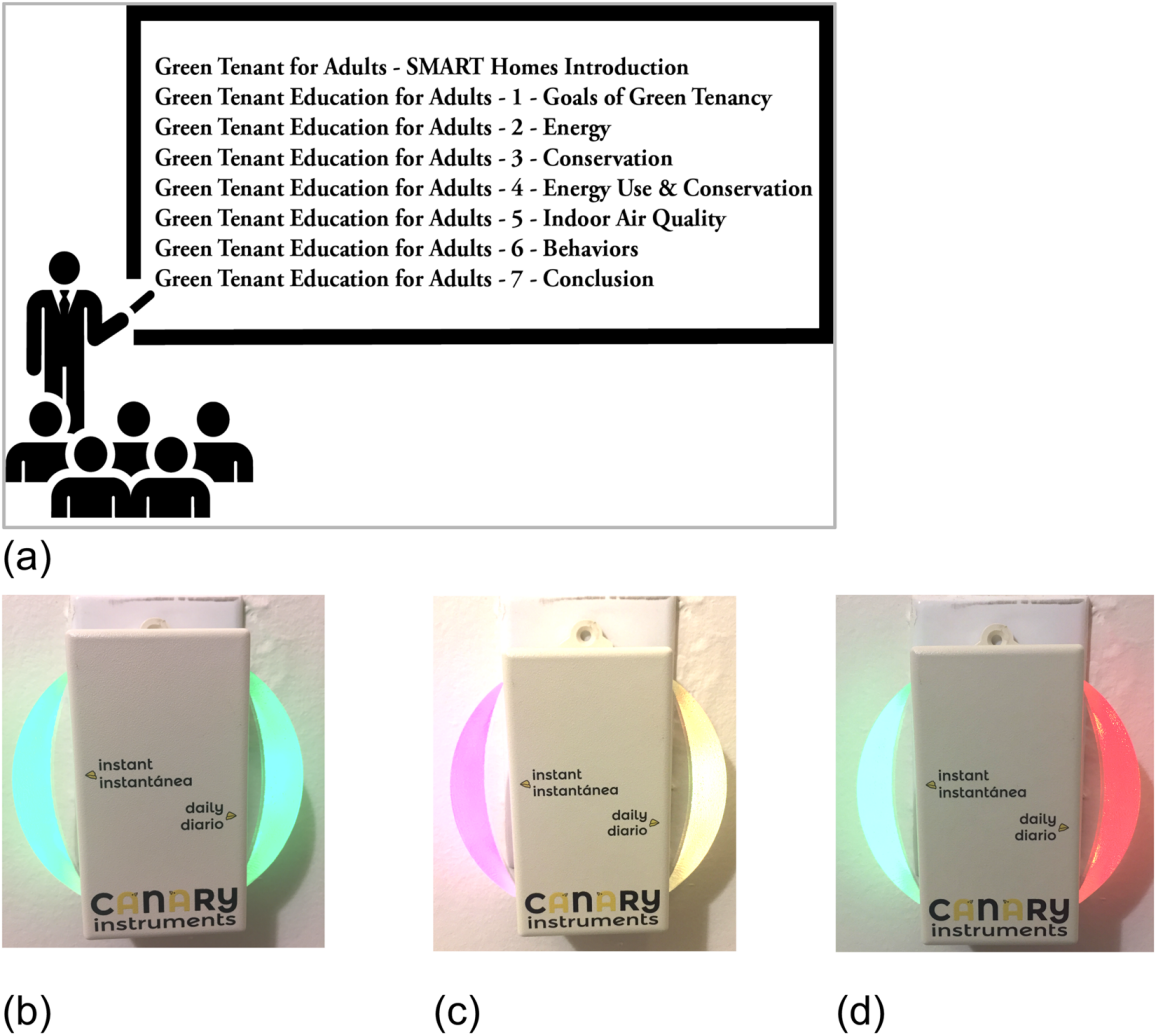
Educational and feedback instruments leveraged in the case study. (**a**) six residents received Targeted Education Intervention (TEI) and an Energy Feedback Display (EFD). The EFD was programmed with a daily energy budget that reflected energy behavior with a dynamic color display; (**b**) EFD interface reset at midnight showing minimal instantaneous use on left with green color, and full daily budget remaining on right with green color; (**c**) high real-time consumption on left with fuchsia color, and 20% of daily budget consumed on right with yellow color; (**d**) low real-time consumption on left with green color, and 70% of daily budget consumed on right with red color.

There are two interventions in this study delivered in the following formats: 1) residents who received a Targeted Energy Intervention (TEI) (see Fig. 1a) on March 28^th^, 2017 and 2) residents who received a TEI + an energy feedback device (see Fig. 1b–d) with the EFD installation occurring on July 6^th^, 2017. The TEI consisted of the authors guiding residents through seven educational videos and a ten-minute PowerPoint presentation that featured technologies specific to their apartment unit.

Principles and concepts in the DOE Energy Literacy guide have a great deal of overlap and the most critical concepts were selected as a scope for this study. Concepts were prioritized in regard to their connection to the TEI and in-home experiences. After the concepts were prioritized, the scope of this study was set to fit the limitations of the data collection and analysis methods utilized. The research team selected five concepts to bound this study which link to daily energy decisions and the targeted energy education we provided: (1) human use of energy is subject to limits and constraints, (2) conservation is one way to manage energy resources, (3) electricity is generated in multiple ways, (4) social and technological innovations affect the amount of energy used by society, and (5) energy use can be calculated and monitored.

The research team considered several factors when developing the daily energy budget for the EFDs. First, the RESNET accredited home energy rating (HERS) energy simulations that were developed during design were reviewed. Second, five years of historical energy use data for the selected apartments were analyzed to develop a measured average kWh/day per apartment. Finally, the team established the energy reduction goals (budgeted kWh/day) to be programmed into the NEXI energy feedback display by multiplying the simulated consumption by the EFD manufacturer’s suggested multiplier for each unit ranging from 1.7 to 1.8. Table 3 provides an overview of the estimated, measured, and budgeted energy goals.

**Table 3:** Energy Feedback Display budgeting.

Description	Unit A	Unit B	Unit C	Unit D	Unit E	Unit F
Simulated kWh/day	3.94	3.94	3.94	3.78	3.62	3.62
Measured kWh/day	7.99	8.96	9.39	11.03	12.07	9.40
Budgeted kWh/day	6.78	6.78	6.78	6.78	6.20	6.20

**Table 4:** Dataset Description.

Sample	Number of Samples	Temporal range	Description	Data
Tabular Data	38 units, 20 Building occupants	July 2013–March 2018	Occupant Demographics, Monthly-time series power data, Pre and Post educational intervention energy literacy, behaviors, and environmental beliefs	flEECe Dataset 5_15_19.xlsx
NEXI data	6 units	July 7, 2017–March-22-2018	Second data at the circuit level for the entire unit, dryer, hot water heater, and HVAC (mini-split) unit	A-F_EST.csv

**Table 5:** Dataset Mapping to Mahdavi and Taheri’s (2017) ontology^19^.

Data Category	Subcategory	Monitored Variables
Energy	Hot Water Heater	Electricity
Energy	Dryer	Electricity
Energy	Heating/cooling (HC)	Electricity
Inhabitants	Attitudes (AD)	Ecological beliefs
Inhabitants	Attributes (AT)	Consumption Behaviors

### Weather and climate data

Weather data for the project location is publicly available through the National Oceanic Atmospheric Administration (NOAA)^23^ and Weather Underground^24^. Historic weather data allows for a researcher to assess the impact of year to year weather variance and climate to weather variances on the site. A baseline of 65 °F/18 °C is used to calculated Heating Degree Day (HDD) and Cooling Degree Day (CDD) with outdoor temperatures measured at the NOAA Richmond International Airport Weather Station (RIAWS). The station is located at 37.51151°, −77.32344° and is approximately 5 miles (8 kilometers) from the case study site. We utilized Weather Underground to retrieve ten years of monthly HDD and CDD from RAIWS.

Next, the team referenced the Typical Meteorological Year (TMY3) data for Richmond, VA, USA to develop a climate benchmark. TMY3 data represents a 30-year average benchmark and was retrieved from the ASHRAE Handbook of Fundamentals^25^. TMY3 data is commonly employed in energy simulation models. The authors leveraged the TMY3 data to unpack 1) simulated performance versus observed performance, 2) yearly weather variance compared to TMY3, and 3) HDD/CDD during the survey period versus the TMY3.

## Data Records

The flEECe dataset can be found on the Open Science Framework^10^. Energy data from each unit was collected utilizing three methods: 1) utility bills in.pdf format, 2) WegoWise utility tracking service, and 3) NEXI energy monitor data loggers. The WegoWise and NEXI data are present in this data set. The utility bills were not stored or shared publicly to protect participant identities. Utility bills were used to spot check the other two records. The WegoWise data is provided in a Microsoft Excel table at a monthly interval (kWh/month/unit). The WegoWise data represents 38 apartments, with 59 contiguous months of data from July 2013 to March 2018. Tables 4 and 5 describe the dataset files and the monitored variables.

The NEXI data is provided as raw (uncalibrated) CSV files and calibrated CSV files. Each CSV has 11 columns. Column A is the timestamp. Columns B-F represent the measured electrical current. These numbers need to be processed through the calibration formula to get amperage values. Column G represents the voltage readings and must be processed through a conversion formula for voltage. A guide for the calibration process is included in the dataset for those who would like develop scripts for managing uncalibrated data from a NEXI device. This will be particularly useful in the future for cloud computing processes using the NEXI’s WIFI capabilities which allow for network connected NEXI’s to upload data directly to a server.

Columns H through L are a representation of phase angle. This was an experimental feature, it may provide some information, but the feature remains in alpha and hasn’t been tested for reliability to the same degree as the Voltage and Current readings. The NEXI calculates time stamps the number of seconds since July 7, 2017. The data provided starts at July 7, 2017 and ends at March 22, 2018.

In the data validation folder of the dataset, timeseries analysis are provided exemplifying one of our processes to ensure the data was valid before advanced analysis and validation processes were executed. It is important to note that there were no single missing seconds, and under a day’s worth of time gaps in the data signifying the NEXI’s were properly recording data consistently as long as they were powered. The identified time gaps most likely reflect power outages as the gaps were registered on multiple independent NEXI devices at the same time. Other data validation processes have been exemplified showing the ability for the data to be disaggregated to discover the use of the dryer at multiple settings.

## Technical Validation

**Fig. 2:**
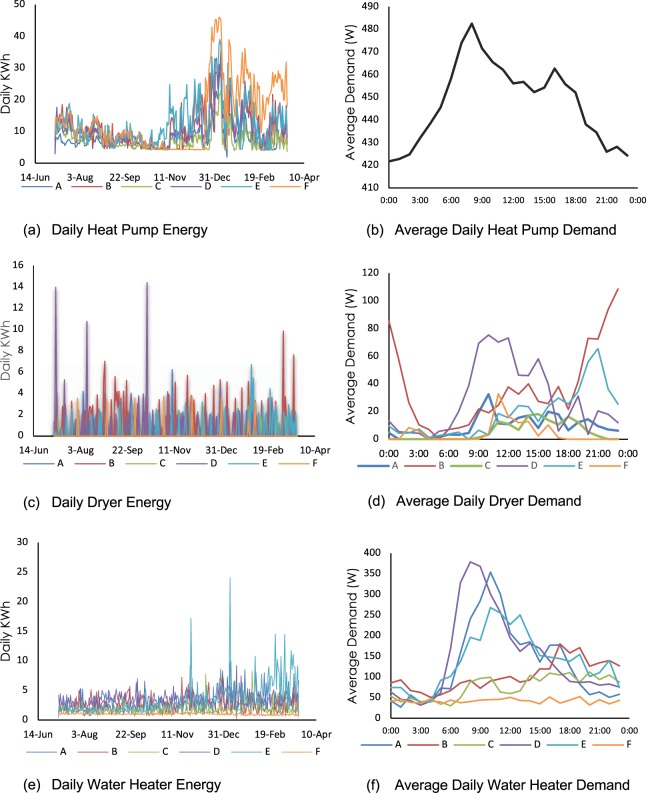
Energy consumption patterns at appliance level. The variations reflect the changes in occupant behavior in these units that were monitored.

In this section, we have presented the results of analyzes on the dataset to ensure that they follow the expected patterns. As shown in Fig. 2, the energy data that was captured at the appliance (sub-circuit) level for the heat pump, dryer, and water heater, manifests rationale variability and reflects the expected patterns in the units and validates the quality of the data. Figure 2a,b shows the variation of energy consumption for the heat pump. Figure 2a shows the variations of daily energy consumption of heat pumps across the period of data collection. The heat pump represents both cooling and heating load variations. As expected, the elevated energy consumption around July and August (representing the cooling load) decreases due to seasonal transition between August and November, where the heating load causes a sharp increase in energy consumption. Furthermore, these graphs show the variability across different units reflecting the diversity in occupant behavior when it comes to operating the loads. Figure 2b shows the average daily variation of the heat pump energy averaged across all units for all days. This graph illustrates the expected variation of energy consumption which reflects the activities of occupants at home. As noted, the occupants of the units are senior citizens and therefore, during the day energy consumption remains relatively high.

On the other hand, the seasonality pattern is not observed in the energy consumption of dryers as shown in Fig. 2c. In other words, the observations show the distributed use of the dryers and the variations across different units. The interactional behavior impact could be better observed in Fig. 2d, in which the differences in dryer use including the time of use and the demand from each unit has been highlighted. The observed variations of the appliance use patterns support the validity of energy use with respect to occupant behavioral patterns across different units.

Furthermore, Fig. 2e,f represent the daily energy consumption and demand of water heaters at different time scales. The former presents the daily variations for the water heater energy consumption across the entire period of data collection. Although most of the units show similar load behavior with relatively constant energy consumption, the occupant behavior impact could be also observed as it has been reflected on the variations of consumption across different days. For unit E the change in consumption behavior could be observed across different months. Although these units are single occupancy units, some units have been occupied by up to two occupants at different times of the data collection period. Figure 2f shows the average daily variation of energy demand for different units. Again, this graph shows the differences in behavior of the occupants in the targeted units and shows reflects the impact of behavioral differences in interacting with loads.

**Fig. 3:**
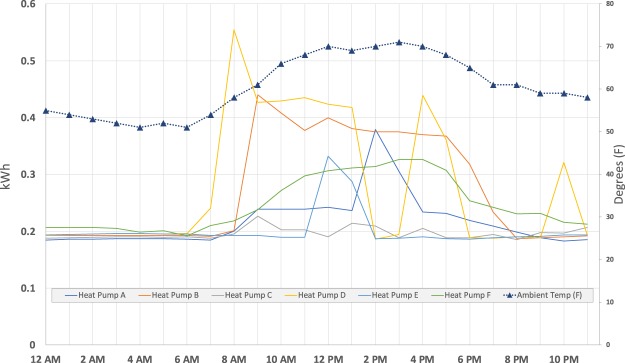
Relationship between heat pump energy consumption and temperature. Sample NEXI data for the 6 units in the flEECe dataset for a day.

Figure 3 illustrates the relationship between energy use for the heat pump circuit during a cooling day. As expected, the heat pumps begin to draw energy as the temperature begins to rise in the morning and loads drop with temperature decline in the evening. The difference in behaviour can also be observed in the energy use.

**Fig. 4:**
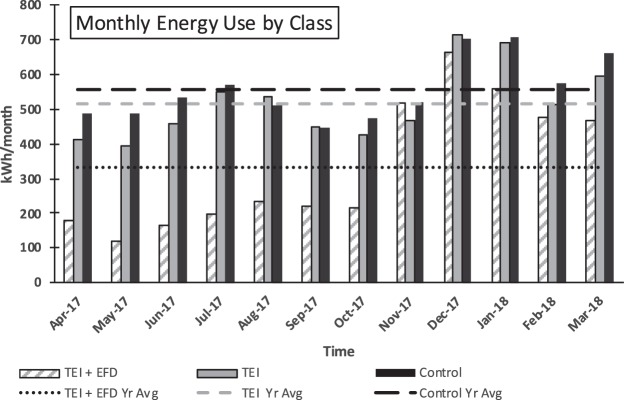
Monthly average energy use (kWh). Representing complete 38 unit sample with interviewed units, TEI units, and control units. Lines represent a yearly average for each group.

Figure 4 illustrates the difference in monthly energy use for the 38 unit sample (from which the final 20 unit survey sample, and 6 unit NEXI sample were selected) over a year. The TEI + EFD units, TEI units, and control units, were compared to analyze the impact of the TEI on energy consumption over a year. The TEI + EFD units average monthly energy consumption was much lower than the TEI units and control group which was expected due to the TEI + EFD units having solar generation offsetting their use.

## Usage Notes

The flEECe dataset is focused around a set of time-series datasets which can be analyzed with a variety of software packages. We encourage the use of Python, which was used in our example files via the Jupyter Notebook Platform to create very accessible HTML files. The use of Plotly is also recommended to create interactive visualizations of the time-series datasets. The fleece dataset also provided access to detailed information about the occupants which to our knowledge has not been combined with timeseries data for seniors in affordable energy efficient housing. The contextual data (perceptions, energy literacy, environmental beliefs), are being prepared for another publication and we encourage users of this dataset to contact the corresponding authors for assistance in leveraging this data. The flEECe dataset will continue to be updated as the longitudinal multiphase study which allowed for the collection of the flEECe dataset is continuing. The authors encourage dialogue from the community to allow for the most useful data to be contributed to the greater scientific community.

## Data Availability

An example of the Python code used to analyze the raw energy monitor data is publicly available on the Open Science Framework data repository^10^. The data can also be analyzed using software which handles tabular timeseries data such as R or MATLAB. The code used to aggregate and plot the data for this paper are publicly available on the data repository on OSF.
